# A Rapid Assessment of the Impact of COVID-19 on Asian Americans: Cross-sectional Survey Study

**DOI:** 10.2196/23976

**Published:** 2021-06-11

**Authors:** Thu Quach, Lan N Ðoàn, Julia Liou, Ninez A Ponce

**Affiliations:** 1 Asian Health Services Oakland, CA United States; 2 Department of Population Health Section for Health Equity New York University Grossman School of Medicine New York, NY United States; 3 Department of Health Policy and Management Fielding School of Public Health University of California, Los Angeles Los Angeles, CA United States; 4 Center for Health Policy Research University of California, Los Angeles Los Angeles, CA United States

**Keywords:** COVID-19, Asian American, testing, mental health, barrier, behavior, impact, discrimination, inequality, disparity, experience, COVID, violence, culture, stress, anti-Asian violence

## Abstract

**Background:**

The diverse Asian American population has been impacted by the COVID-19 pandemic, but due to limited data and other factors, disparities experienced by this population are hidden.

**Objective:**

This study aims to describe the Asian American community’s experiences during the COVID-19 pandemic, focusing on the Greater San Francisco Bay Area, California, and to better inform a Federally Qualified Health Center’s (FQHC) health care services and response to challenges faced by the community.

**Methods:**

We conducted a cross-sectional survey between May 20 and June 23, 2020, using a multipronged recruitment approach, including word-of-mouth, FQHC patient appointments, and social media posts. The survey was self-administered online or administered over the phone by FQHC staff in English, Cantonese, Mandarin, and Vietnamese. Survey question topics included COVID-19 testing and preventative behaviors, economic impacts of COVID-19, experience with perceived mistreatment due to their race/ethnicity, and mental health challenges.

**Results:**

Among 1297 Asian American respondents, only 3.1% (39/1273) had previously been tested for COVID-19, and 46.6% (392/841) stated that they could not find a place to get tested. In addition, about two-thirds of respondents (477/707) reported feeling stressed, and 22.6% (160/707) reported feeling depressed. Furthermore, 5.6% (72/1275) of respondents reported being treated unfairly because of their race/ethnicity. Among respondents who experienced economic impacts from COVID-19, 32.2% (246/763) had lost their regular jobs and 22.5% (172/763) had reduced hours or reduced income. Additionally, 70.1% (890/1269) of respondents shared that they avoid leaving their home to go to public places (eg, grocery stores, church, and school).

**Conclusions:**

We found that Asian Americans had lower levels of COVID-19 testing and limited access to testing, a high prevalence of mental health issues and economic impacts, and a high prevalence of risk-avoidant behaviors (eg, not leaving the house) in the early months of the COVID-19 pandemic. These findings provide preliminary insights into the impact of the COVID-19 pandemic on Asian American communities served by an FQHC and underscore the longstanding need for culturally and linguistically appropriate approaches to providing mental health, outreach, and education services. These findings led to the establishment of the first Asian multilingual and multicultural COVID-19 testing sites in the local area where the study was conducted, and laid the groundwork for subsequent COVID-19 programs, specifically contact tracing and vaccination programs.

## Introduction

COVID-19, a respiratory disease caused by SARS-CoV-2, was first identified in China in December 2019, and then quickly spread throughout the world. By March 2020, COVID-19 became a global pandemic, devastating many countries, including the United States. In California, many health care organizations have had to rapidly modify their services to telehealth and remote services in response to shelter-in-place orders and to minimize the spread of the virus. At the same time, Asian Americans have reported increased discrimination since the beginning of the outbreak due to anti-Asian rhetoric related to the virus originating in China [1].

The Asian American population is diverse in language, culture, and immigration history, which affects their experiences during the COVID-19 pandemic. Due to language barriers and limited data collection, disparities for this population often remain hidden [2]. During this pandemic, these issues may become exacerbated, and this population may run the risk of suffering in silence. Data from different geographic areas suggest that Asian Americans have high death rates among those who test positive for COVID-19 [3,4]. A study by the Kaiser Family Foundation of 50 million patients in the Epic health records system also found that Asian American patients, along with Black and Hispanic patients, have significantly higher rates of hospitalization and death due to COVID-19 compared to their White counterparts. However, there was little attention to COVID-19–related health disparities faced by Asian Americans [5].

Asian Health Services (AHS), a Federally Qualified Health Center (FQHC), provides medical, dental, behavioral health, and health education/outreach services in 14 languages to approximately 50,000 patients in Alameda County, California. When the pandemic emerged in March 2020, AHS quickly shifted to telehealth and remote services to minimize the risk of spreading the COVID-19 virus. Many AHS patients began to stay at home even before the state released a shelter-in-place order, and the streets of local areas with a high density of Asian Americans (eg, Oakland Chinatown) became empty. As in other Chinatowns and ethnic enclaves nationally [6] (Russo et al, unpublished data, 2020), many restaurants and other small businesses closed down. When AHS staff called to connect with patients via telehealth and general check-in appointments, they heard hundreds of patient stories about ongoing anti-Asian attacks and discrimination incidents, increased fear and isolation as many stayed in their homes, and growing mental health issues, including suicide attempts and domestic violence. As part of a rapid response to these patient narratives, AHS implemented telehealth services (medical, mental health, and dentistry) and continued to provide high-quality care throughout the COVID-19 pandemic, while concurrently responding to rising social needs, sounding the alarm on anti-Asian racism, and advocating for better data collection and disaggregation.

While California had implemented a shelter-in-place order, AHS observed that many patients and those in the local community were not coming out of their homes, even for necessities (eg, grocery shopping) and other essential services (eg, preventative health care appointments). At the same time, AHS observed low testing rates in Asian American communities, and suspected these low rates were due to barriers including language access, fear and stigma due to the rising anti-Asian landscape [7], and lack of information on resources. The purpose of this study was to describe the Asian American community’s experiences during the COVID-19 pandemic, focusing on the Greater San Francisco Bay Area in California, and to better inform health care services provided at AHS.

## Methods

We conducted a cross-sectional survey between May 20 and June 23, 2020, using a multipronged recruitment approach, including word-of-mouth through AHS social networks, the AHS patient pool (eg, wellness check-ins), and social media posts (eg, Facebook, WeChat). The survey was self-administered online or administered over the phone by AHS staff in English, Cantonese, Mandarin, and Vietnamese. The online survey was available in English, traditional Chinese, Vietnamese, and Korean (although we did not have any Korean respondents). The surveys were translated and reviewed by a certified translation vendor and then additionally reviewed by trained and experienced AHS staff, who are familiar with our target population. The 20-question survey included respondent demographics, COVID-19–related testing and preventative behaviors, economic impacts of COVID-19, experience with anti-Asian violence, and mental health challenges (Multimedia Appendix 1). The questions were mostly closed-ended. There was one open-ended question that allowed the respondent to share their experiences with “discrimination or violence (verbal, emotional, or physical)” due to their race/ethnicity. The survey instrument was designed by AHS health care staff, including several immigrant health researchers and a chief medical officer, and was pilot tested by four patients, two of whom had limited English proficiency. We used five questions from the California Health Interview Survey (CHIS), as the questions have been tested and validated [8]. CHIS is a web-based and telephone interview survey of over 20,000 Californian adults, teenagers, and children, and it is representative of the 58 counties in the state.

The survey included responses from individuals aged ≥12 years. We decided to use this age cutoff because AHS has several youth programs, which serve youth as young as 12 years of age. The AHS youth program staff conducted additional outreach to adolescents and young teens to understand the needs and experiences of Asian American youth during COVID-19.

This study was designed to be descriptive in design, so as to document and understand the COVID-19 experiences and behaviors of the AHS patient population, in order to inform the development of intervention and public response programs (eg, COVID-19 community testing sites). Our study is exempt from institutional review board (IRB) approval because we did not collect any personally identifiable information and the main purpose of the study was to examine FQHC health care services (ie, a public benefits program design rather than a research study); thus, we did not obtain written consent from participants. For the telephone-administered surveys, AHS staff verbally asked if patients were willing to voluntarily participate in the survey before administering the questions. For the online surveys, respondents voluntarily completed the survey and we emphasized in the survey text that the survey is voluntary and anonymous, and that we would not collect any personally identifiable information. We used descriptive statistics to summarize respondent characteristics and responses to the COVID-19 questionnaire.

## Results

We surveyed 1279 individuals over a one-month period. The majority of respondents were women (760/1273, 59.7%), Chinese (1127/1278, 88.2%), foreign-born (1020/1254, 81.3%), and not fluent in English (718/1279, 56.1%); in addition, most participants resided in Oakland (586/1113, 52.7%; Table 1).

Only 3.1% (39/1273) were tested and there was a 5% (2/39) positivity rate among respondents who were tested (Table 2). The primary reasons that respondents reported for not getting tested were not finding a testing site (392/841, 46.6%) and not being concerned that they had been exposed to the virus (355/841, 42.2%). Respondents reported wearing a mask before (503/1270, 39.6%) and after (519/1270, 40.9%) it became required by the government. In addition, 70.1% (890/1269) reported avoiding leaving their house (eg, going to the grocery store, church, and school) to avoid COVID-19 infection.

Among respondents who reported experiencing economic impacts from COVID-19, 32.2% (246/763) reported losing their regular jobs and 22.5% (172/763) reported reduced hours or reduced income. About 12.7% (97/763) of respondents had financial difficulties related to paying their rent or mortgage and for basic necessities (eg, paying bills and tuition, affording groceries).

In terms of the mental health impact of the COVID-19 pandemic, 67.5% (477/707) of respondents reported feeling stressed, 22.6% (160/707) reported feeling depressed, and 15.8% (112/707) reported feeling restless or fidgety. Furthermore, 5.6% (72/1275) of respondents reported being treated unfairly because of their race/ethnicity, which was greater than the state average during this time period [9]. Among respondents who reported experiencing unfair treatment, only one person reported the incident.

For the open-ended question on “discrimination and violence,” there were 24 respondents who wrote comments. Most of the comments were about being yelled at or being given “dirty looks” for carrying the virus. One respondent shared, “I was called, ‘corona china’ from a random person and was told that from a cashier that Korean people are coming here with the virus. I reduced going out after these incidents.” Other respondents shared, “people have yelled at me while I am wearing a mask” or “people run when they see me wearing a mask.” Another respondent shared, “just going grocery shopping with my parents we always get mugged. Even in line to pay a woman had the audacity to call us racial slurs.”

**Table 1:** Demographic characteristics of Asian Health Services survey respondents, Greater San Francisco Bay Area, May-June 2020 (N=1279).

Characteristic			Particpants, n (%^a^)
**Gender (n=1273), n (%)**			
	Male	513 (40.3)	
	Female	760 (59.7)	
**Race/ethnicity (n=1278), n (%)**			
	Cambodian	20 (1.6)	
	Chinese	1127 (88.2)	
	Vietnamese	103 (8.1)	
	Other Asian	20 (1.5)	
	Two or more races	8 (0.6)	
**Age groups, years (n=1141), n (%)**			
	12-18	13 (1.1)	
	18-24	332 (29.1)	
	25-44	222 (19.4)	
	45-64	323 (28.3)	
	≥65	251 (22.0)	
**Birthplace (n=1254), n (%)**			
	United States	234 (18.7)	
	Foreign born	1020 (81.3)	
Number of people per household (n=1278), average (SD)			3.5 (1.6)
**English fluency (n=1279), n (%)**			
	Fluent or speak pretty well	388 (30.3)	
	Speak somewhat well	173 (13.5)	
	Do not speak English very well or at all	718 (56.1)	
**Residence (n=1113), n (%)**			
	Oakland	586 (52.7)	
	San Leandro	202 (18.1)	
	Alameda	79 (7.1)	
	San Francisco	41 (3.7)	
	Other cities in Alameda County	143 (12.8)	
	Other cities	62 (5.6)	

^a^Percentages are based on nonmissing numbers.

**Table 2:** COVID-19 testing and preventative behaviors and COVID-19 impact on Asian Health Services survey respondents, Greater San Francisco Bay Area, May-June 2020.

Question		Participants, n (%^a^)
**Have you ever had or thought you might have had COVID-19?**		
	Yes	47 (3.7)
	No	1229 (96.3)
	Don't know/refused to answer	3
**Were you ever tested for COVID-19?**		
	Yes	39 (3.1)
	No	1234 (96.9)
	Don't know/refused to answer	6
**What was the reason why you did not get tested?**		
	I was not able to find a place that would test me.	392 (46.6)
	I was told by a health professional that I did not need to get tested.	37 (4.4)
	I was afraid that a positive test would require me to get health care, and I was worried about the cost.	20 (2.4)
	I was afraid that using health care could affect my immigration status.	6 (0.7)
	I was afraid of being discriminated against if others knew I was positive.	5 (0.6)
	I thought if I could just isolate myself in my home, I would get better and not infect other people.	35 (4.1)
	I was not concerned that I had been exposed to the virus.	355 (42.2)
	Don't know/refused to answer	438
**Did you ever receive a positive test result for COVID-19?**		
	Yes	2 (0.2)
	No	1275 (99.8)
	Don't know/refused to answer	2
**Has anyone in your household that you live with ever tested positive for COVID-19?**		
	Yes	10 (0.8)
	No	1252 (99.2)
	Don't know/refused to answer	17
**Have you experienced any of the following situations because of the COVID-19 outbreak?**		
	I have lost my regular job.	246 (32.2)
	I have had a reduction in hours, or a reduction in income.	172 (22.5)
	I have switched to working from home.	121 (15.9)
	I have continued to report to work because I was an essential worker.	88 (11.5)
	I have had difficulty in obtaining childcare, or had an increase in childcare expenses.	53 (6.9)
	I have had financial difficulties with paying the rent or mortgage.	92 (12.1)
	I have had financial difficulties with basic necessities, such as paying bills or tuition, affording groceries, etc.	97 (12.7)
	I have been treated unfairly because of my race/ethnicity.	41 (5.4)
	I have experienced other challenges (Specify: ______)	7 (0.9)
	No response or none of the above	516
**Have you had any experiences of discrimination or violence (verbal, emotional, or physical) due to your race/ethnicity during this COVID-19 outbreak?**		
	Yes	72 (5.6)
	No	1203 (94.4)
	Don't know/refused to answer	4
**Have you reported your experience?**		
	Yes	1 (1.4)
	No	71 (98.6)
	Don't know/refused to answer	1207
**Since the COVID-19 outbreak began, have you felt any of the following?**		
	Depressed	160 (22.6)
	Hopeless	45 (6.4)
	Stressed	477 (67.5)
	Restless or fidgety	112 (15.8)
	Other	12 (1.7)
	Don't know/refused to answer	572
**Have you talked to your doctor or a mental health professional about how you felt?**		
	Yes	69 (5.5)
	No	1196 (94.5)
	Don't know/refused to answer	14
**Please indicate how long you have been wearing a mask.**		
	Wore a mask before shelter-in-place	503 (39.6)
	Wore a mask when shelter-in-place started	519 (40.9)
	Wore a mask after the government required us to do so	243 (19.1)
	I do not wear a mask in public.	5 (0.4)
	Don't know/refused to answer	9
**Have you done other things to reduce your chances of getting infected with COVID-19?**		
	Avoid leaving my house to go to any public places (such as grocery stores, church, and school)	890 (70.1)
	Avoid going to any of my health care appointments	105 (8.3)
	Avoid taking public transportation	238 (18.8)
	Other	36 (2.8)
	Don't know/refused to answer	10

^a^Percentages are based on nonmissing numbers and definitive answers (ie, responses of “don’t know” and no response were not included in the total).

## Discussion

The survey results underscore the different and interconnected needs of and issues in the Asian American community during the early stages of the pandemic. We found that Asian Americans reported low levels of COVID-19 testing (3%) and limited access to testing, high prevalence of mental health issues and economic impacts, and high prevalence of risk-avoidant behaviors (eg, not leaving the house) in the early months of the COVID-19 pandemic.

A high percentage of respondents reported they did not leave their homes, which likely reduced their risk of COVID-19 infection. However, another possible explanation for not leaving the home may be increased fear of unprovoked anti-Asian hate, harassment, and discrimination. Overall, 6% of respondents reported experiencing discrimination or violence. The quotes from some respondents also highlight the anti-Asian sentiment. The survey was conducted 2-3 months into the pandemic, relatively early on in the pandemic. We anticipate the prevalence of these experiences would increase over time. The data from this survey is consistent with the growing number of studies and reports on the rise in anti-Asian discrimination during the pandemic, which often involves physical violence and harassment [10-14].

The prevalence of perceived unfair treatment may also explain the high prevalence of self-reported mental health issues. Other studies have demonstrated the association between the increase of anti-Asian discrimination and poorer mental health among Chinese Americans and Asian Americans [10,15]. Within AHS, our mental health providers have shared that there is an increase in mental health utilization since the pandemic started. For example, our mental health appointments had previously typically not been fully booked, but since the pandemic started, our mental health appointments have been fully booked, and there was a decline in no-shows for appointments from 20% to close to zero. AHS mental health providers have noted that patients have shared their experiences of fear and trauma relating to both COVID-19 and anti-Asian hate, causing many patients to become fearful of leaving their homes. The social isolation experienced may also contribute to mental health impacts.

Our results highlight the low testing rate among AHS Asian American patients (3%). In comparison to testing rates in Alameda County during the same time period, where a majority of respondents reside, the testing rate across all racial groups was 174.3 per 1000 (17.4%), across all races, while the testing rate for Asian Americans was the lowest at 57.73 per 1000 (5.8%) [16]. Similarly, the Kaiser Family Foundation found COVID-19 testing rates were lower among Asian patients compared to White patients [5]. Furthermore, nearly half of respondents who did not get a test reported that they were not able to find a place to get tested. During the survey period, COVID-19 tests were difficult to access and even more difficult to access in Asian languages. Based on our work with this patient population and the broader literature base on Asian American health disparities, we hypothesize that language access was a major barrier to access to testing for Asian Americans and will remain a large barrier during the COVID-19 vaccination process if left unaddressed.

Our finding that the low testing rates were due to limited access to testing sites provided us with important data that we presented to our local government to justify the need for multilingual (eg, Cantonese, Mandarin, Vietnamese, Korean, Khmer, Tagalog, Burmese, Tongan, and Samoan) COVID-19 testing sites situated within neighborhoods with a higher density of Asian Americans (eg, Oakland Chinatown and Little Saigon of Oakland). Given the results of this survey, AHS has established several COVID-19 community testing sites that provide culturally appropriate services in multiple languages at several locations in Oakland and Fremont that have a higher density of Asian Americans. In addition, AHS launched a multilingual helpline to provide information and navigation assistance regarding the COVID-19 testing process, social services (eg, food and unemployment assistance), consultations for isolation and quarantine, and other case management and mental health referrals. Since the establishment of these services, the number of Asian Americans who receive COVID-19 testing has increased, and AHS has observed the successful use of the multilingual helpline to obtain services. The findings have also informed the establishment of a culturally and linguistically competent contact tracing program and vaccine rollout processes. AHS has seen high uptake of these services, including testing, contact tracing connections, and vaccinations, particularly among Asian American individuals with limited English proficiency; this is likely because AHS services have been developed and tailored to be culturally, linguistically, and geographically relevant, and are situated in neighborhoods where there are higher numbers of Asian Americans to increase access to these resources.

These findings are informative for other FQHCs serving similar populations, including the 32 community health centers across the nation within the Association of Asian Pacific Community Health Organization (AAPCHO), a network of community health organizations serving tens of millions of low-income Asian Americans as well as Native Hawaiian and Pacific Islanders across the country.

There are some limitations to our study. First, we cannot be certain that no individuals took the survey multiple times since we did not collect personally identifiable information. However, we did not provide any incentives for participation and do not believe there was any reason for respondents to take the survey multiple times. We also did not collect information that distinguishes respondents that completed the survey online from respondents who completed the survey administered by AHS staff. For the surveys that were administered by AHS staff, there is a potential for social desirability and misreporting. Thus, there is a possibility that there may be some differences in responses to these two methods of survey administration. Additionally, this survey was conducted at the beginning of the pandemic and for the purposes of improving FQHC services for a public benefits program design; therefore, we did not used a validated survey instrument. However, the development of survey questions was guided by health researchers and AHS staff familiar with the patient population, as well as questions from the CHIS, the largest state health survey in the United States. Furthermore, the survey was pilot tested by four patients who confirmed that questions were straightforward for English speakers and those with limited English proficiency and that the survey was feasible to complete during patient appointments. We did not use standardized discrimination questions because those instruments have not been well tested among Asian American populations with limited English proficiency. The COVID-19 pandemic is unprecedented in multiple ways, including the global morbidity and mortality impact, as well as the fact that Asian American communities have been targeted and blamed for causing the virus. At the time of our survey, there were no survey instruments that had been designed and tested to capture discrimination experiences during a pandemic. As health care providers who have served and advocated for the Asian American population since 1974, we designed questions that would allow us to capture our patients’ experiences while being easy to comprehend and complete.

The survey findings highlight the ongoing need to use community-based approaches, including culturally and linguistically competent survey instruments, to document emerging issues in vulnerable populations to inform care and implement strategies to address disparities. At the time of our survey, there were no existing data to document COVID-19–related experiences among Asian Americans, and few peer-reviewed studies have been published on the Asian American experience one year into the pandemic [3,4,17,18]. As trusted health care providers who were hearing from hundreds of patients about the fear of infection and anti-Asian discrimination, the mental health impacts, and the lack of knowledge about resources due to language barriers, AHS implemented this survey, which informed our rapid response to COVID-19. The AHS experience can serve as a successful case study of evidence-based COVID-19 response efforts for other Asian American–serving FQHCs. The data on low testing, limited access to testing, and high mental health needs resulted in the establishment of culturally and linguistically competent COVID-19–related services (eg, testing, contact tracing, and vaccinations) and increased the overall utilization of the health services among Asian Americans during the pandemic.

## Appendix

Multimedia Appendix 1 Survey instrument.

## Abbreviations

AHS: Asian Health Services
CHIS: California Health Interview Survey
FQHC: Federally Qualified Health Center
IRB: Institutional Review Board
