# Supplementary material for: A Rapid Assessment of the Impact of COVID-19 on Asian Americans: Cross-sectional Survey Study
Source: JMIR Public Health Surveill. 2021 Jun 11;7(6):e23976. doi: 10.2196/23976 (PMC8202653; doi:10.2196/23976)
Supplement: Multimedia Appendix 1 [file publichealth_v7i6e23976_app1.docx]

**Supplementary Online Content**

**eMethods.** COVID-19 Survey Instrument

## COVID-19 Community Survey

Draft: 5/17/2020

**Asian Health Services (AHS)** is conducting a quick survey about COVID-19 (also known as the Coronavirus). The results of the survey will inform AHS, our partners, and policy-makers on the needs in our community. We appreciate your participation in this survey, which is expected to take about 15 minutes. All responses will be kept confidential. The survey is anonymous, which means we will not collect any information that would identify who you are.

1. **Are you male or female?**

- Male
- Female
- Do not identify as male or female (non-binary)
- Refused to disclose

1. **What racial/ethnic group do you identify with?** *[Select all that apply]*

- Asian *[Select all that apply]*
  - Asian Indian
  - Burmese
  - Cambodian
  - Chinese
  - Filipino
  - Hmong
  - Japanese
  - Korean
  - Laotian
  - Mongolian
  - Thai
  - Vietnamese
  - Other (specify: ________)
- Pacific Islander*[Select all that apply]*
  - Native Hawaiian
  - Guamanian
  - Samoan
  - Tongan
  - Other (specify: ________)
- African American/ Black
- American Indian/ Alaska Native
- Hispanic/ Latinx
- Non-Hispanic White
- Other: _________________

1. **How old are you?** _____YEARS OF AGE
2. **Where were you born?** _________________________
3. **What is your residential zip code (where you live)?** ________________
4. **How many people live in your household?** _____________
5. **How well do you speak English?**

- Fluently like a native
- Speak pretty well
- Speak somewhat well or so so
- Not very well
- Not at all

1. ***Have you ever had, or thought you might have had, the Coronavirus, COVID-19?**
   - Yes
   - No
2. ***Were you ever tested for COVID-19?**
   - Yes (*If yes, go to Q11)*
   - No
3. If No, what were the reasons why you did not get tested? [Select all that apply]

- I was not able to find a place that would test me.
- I was told by a health professional that I did not need to get tested.
- I was afraid that a positive test would require me to get health care, and I was worried about the cost.
- I was afraid that using health care could affect my immigration status.
- I was afraid of being discriminated against if others knew I was positive.
- I thought if I could just isolate myself in my home, I would get better and not infect other people.
- I was not concerned that I had been exposed to the virus.
- Other: (Please specify: __________________________)

1. ***Did you ever receive a positive test result for COVID-19?**
   - Yes
   - No (*If no, go to Q13)*
2. ***Were you ever a patient in a hospital overnight or longer because of COVID-19?**
   - Yes
   - No
3. **Has anyone in your household that you live with ever been tested positive for COVID-19?**
   - Yes
   - No
4. ***Have you experienced any of the following situations because of the**

**Coronavirus or COVID-19 outbreak**? *[Select all that apply]*

- I’ve lost my regular job.
- I’ve had a reduction in hours, or a reduction in income.
- I’ve switched to working from home.
- I’ve continued to report to work because I was an essential worker.
- I’ve had difficulty in obtaining childcare, or had an increase in childcare expenses.
- I’ve had financial difficulties with paying rent or mortgage.
- I’ve had financial difficulties with basic necessities, such as paying bills, tuition, affording groceries, etc.
- I’ve been treated unfairly because of my race/ethnicity.
- I’ve experienced other challenges (Specify:______)

1. **Have you had any experiences of discrimination or violence (verbal, emotional, or physical) due to your race/ ethnicity during this COVID-19 outbreak?**

- Yes (Please share more if you are comfortable: ________________________________)
- No *(If no, go to Q17)*

1. **Have you reported your experience to the police or to any online reporting sites?**

- Yes (Please share where: ________________________________________)
- No (Please share why: __________________________________________)

1. **Since Covid-19 outbreak began, have you felt any of the following?** *[check all that apply]*

- Depressed
- Hopeless
- Stressed
- Restless or fidgety
- Other (Please specify: ________________________________________)

1. **Have you been able to talk to your doctor or a mental health professional about how you felt?**

- Yes
- No

1. **Wearing masks in public can reduce the risk of being infected or infecting others. Please indicate how long you have been wearing a mask.**

- I have been wearing a mask in public, even before shelter-in-place.
- I have been wearing a mask when shelter-in-place started.
- I have been wearing a mask in public after the government required us to do so.
- I do not wear masks in public.

1. **Have you done other things to reduce your chances of getting infected with COVID-19?**

- Avoid leaving my house to go to any public places (such as grocery stores, church, and school).
- Avoid going to any of my health care appointments.
- Avoid taking public transportation.
- Other (Specify: ______________________)

**Indicates California Health Interview Survey (CHIS) question*
